# Neurocognitive characterization and academic impact in pediatric patients belonging to the national registry of GA-1

**DOI:** 10.1186/s13023-025-03668-6

**Published:** 2025-12-29

**Authors:** Zamora-Crespo Berta, Moreno-Ramos Zaida, Martínez de Aragón Ana, Núñez-Enamorado Noemí, Martin-Bejarano Manuela, Villaluenga Raquel, Barrio Delia, Bellusci Marcello, Martín-Hernández Elena, Quijada-Fraile Pilar

**Affiliations:** 1 Neuropsychology Department, Pediatric Service, Hospital Universitario 12 de Octubre, Avda. de Córdoba s/n, 28041 Madrid, Spain; 2 Radiology Department, Pediatric Service, Hospital Universitario 12 de Octubre, Avda. de Córdoba s/n, 28041 Madrid, Spain; 3 Neurology Department, Pediatric Service, Hospital Universitario 12 de Octubre, , Avda. de Córdoba s/n, 28041 Madrid, Spain; 4 Hereditary Mitochondrial Metabolic Diseases Department, Pediatric Service, Hospital Universitario 12 de Octubre, Avda. de Córdoba s/n, 28041 Madrid, Spain

**Keywords:** Glutaric aciduria type 1 (GA-1), Neurocognitive characterization, Pediatric patients, Learning disorders, Neurodevelopmental impact

## Abstract

**Background:**

Glutaric Aciduria Type 1 (GA-1) is a rare metabolic disorder characterized by a deficiency in glutaryl-coenzyme A dehydrogenase (GDH), leading to the accumulation of neurotoxic compounds that affect neurodevelopment. This study investigated the neurocognitive profiles and academic challenges faced by pediatric patients with GA-1. To explore the neurocognitive characterization and academic impact in pediatric patients with GA-1 from the National Registry of GA-1.

**Methods:**

This prospective, observational, multicenter study included 42 pediatric patients (25 boys and 17 girls) from a national registry. Neurocognitive evaluations were performed using age-appropriate psychometric tests. Data analysis included analysis of variance (ANOVA) and random forest models to identify the neurocognitive variables that impact learning outcomes.

**Results:**

The patients showed significant variability in neurocognitive outcomes. Children under 4 years of age had average cognitive development and deficits in gross motor skills. Older children had average intelligence scores but moderate to severe impairments in executive functions, attentional processes, and visuocognitive skills. Approximately 60% of the participants required special educational support.

**Conclusions:**

GA-1 patients exhibit neurocognitive impairments that affect learning, necessitating personalized educational interventions. Therefore, early diagnosis and management of this condition are critical**.** Further research is needed to explore long-term neurocognitive outcomes and the relationship between biochemical subtypes and clinical outcomes.

## Introduction

Glutaric aciduria type I (OMIM #231670), a rare metabolic disorder caused by mutations in the GCDH gene, was first described in 1975 [10]. Its prevalence is one in 100,000 newborns [15, 16]. This disease is characterized by a deficiency in glutaryl-coenzyme A dehydrogenase (GDH), which is responsible for the dehydrogenation and decarboxylation of glutaryl-CoA in the degradation pathway of lysine, hydroxylysine, and tryptophan [8]. These acidic compounds are highly toxic to organisms, especially the nervous system; therefore, they are considered neurotoxic (“Glutaric aciduria type I,” 2014).

In relation to the neurodevelopment of children diagnosed with Inherited Metabolic Diseases (IMD), there is scarce literature on the subject, although it is known that metabolic diseases lead to severe disabilities in many patients, which significantly affect their quality of life [6]. Given the characteristics of brain alterations in GA1, white matter changes, basal ganglia injury, frontotemporal cortical hypoplasia, and cumulative neurotoxicity, these patients are at high risk for neurological and neurocognitive damage [13]. Even patients undergoing treatment from an early age with a lisine-restricted diet, emergency regimen, and drugs that favor the excretion of glutaric acid and its derivatives show frequent neuroradiological [12] and neurocognitive alterations.

Patients with GA-1 are classified into high excretion (HE) and low excretion (LE) groups based on the urinary levels of glutaric acid and 3-hydroxyglutaric acid in their urine. High excreters typically have elevated levels of these acids, with glutaric acid levels exceeding 100 mmol/mol creatinine, and 3-hydroxyglutaric acid levels above 5 mmol/mol creatinine. In contrast, the low excreters had significantly lower levels of these metabolites. Recently, Märtner et al. [21] published a study on the biochemical subtypes of GA-1, specifically focusing on patients with high excretion (HE) and low excretion (LE) as predictors of cognitive function. Considering the clinical, biochemical, sociodemographic, and cognitive variables, they studied the role of GA-1 biochemical markers in neurocognitive development. The study found that patients with the HE tended to have worse cognitive performance than those with the LE.

In 2019, a national registry for GA-1, which is a crucial resource for understanding the pathogenesis of the disease and its impact on the central nervous system, was established. This initiative aimed to bring the expertise of multiple specialists into a collaborative platform to identify the variables that negatively impact neurodevelopment. This study aimed to investigate the impact of glutaric aciduria type 1 on neurodevelopment and learning in pediatric patients to identify the risk factors for the disease, thus preventing long-term neurocognitive sequelae.

## Material and methods

### Study design

This prospective observational multicenter study included 42 patients (25 boys and 17 girls). All the patients were enrolled in a Spanish national registry managed by the Pediatric Service of the Hospital Universitario 12 de Octubre (http://redcap.imas12.es).

Patients were recruited from various hospitals across 13 autonomous communities that participated in the study. The inclusion criteria were (a) age between 0 and 16 years, (b) diagnosis of AG-1, and (c) written informed consent from the parents. Patients who did not fulfill these criteria were excluded from the study.

This study was approved by the Research Institute of Hospital, 12 de Octubre, Madrid. Data were collected after obtaining written informed consent from GA-I patients or their parents as well as from control subjects. The data were then pseudonymized prior to the statistical analysis.

### Outcome parameters

This study collected data on socioeconomic, clinical, educational, neurocognitive, and socioemotional variables. The socioeconomic data provided relevant information on age, educational level, economic status, and employment status. The neurocognitive data collected included cognitive, attentional, executive, motor, and communicative functions by means of psychometric tests, which were standardized in the Spanish population and referenced in national and international scientific literature.

Cognitive outcomes, development, and cognition were evaluated using the age-dependent developmental and intelligence tests (Table 1).

**Table 1:** Assessment tools and cognitive domains evaluated by age group: This table outlines the neuropsychological tests administered across different age groups, highlighting the cognitive domains assessed, including intelligence, executive functions, attention, and motor skills

Age group	Tests	Cognitive domain
Up to 4 years	Bayley Development Scale III	General cognitive development
Up to 4 years	Kaufman’s IQ Test	Fluid reasoning, crystallized intelligence, total IQ
Up to 4 years	Rey-Osterrieth Complex Figure (ROCF)	Visuocognitive processes
4 years and older	Digit Span Backward, Digit Span Sequencing, Coding (WISC)	Attention
4 years and older	Gray and Colored Sets/TMT Part A and B (ENFEN)	Executive functions
4 years and older	Grooved Pegboard	Motor skills
All patients	Coding (WAIS-IV) and TMT Part A/Gray Sets	Processing speed
All patients	Educational Context	Regular schooling with support or Special educational needs with adaptations

*KABC* Kaufman assessment battery for children, *ROCF* Rey–Osterrieth complex figure, *TMT* trail making test, *ENFEN* evaluación neuropsicológica de las funciones ejecutivas en niños, *WISC* Wechsler intelligence scale for children, *WAIS-IV* Wechsler adult intelligence scale—fourth edition

### Statical analysis

Python was used to perform the statistical analysis in combination with the following specialized libraries: SciPy: Used for statistical analysis and modeling [27], matplotlib and Seaborn: Used to create graphs and visualizations [14, 28], Numpy: Fundamental for numerical data manipulation [11], Pandas: Facilitated the manipulation of data frames [22], Pingouin: Used for additional statistical testing and post hoc analysis [26]. These tools provide a complete approach for statistical analysis and data visualization in this study. Data were collected, including sample information and mean, median, standard deviation (SD), interquartile range (IQR), and full range (Table 1). In the analysis of cognitive outcomes, independent variables related to neurological manifestations were assessed, including encephalopathy, seizures, and lesions detected using magnetic resonance imaging (MRI). Independent variables related to neurocognitive studies were considered in the analysis of the learning disorders.

Raw scores for all psychological tests were converted to standard deviation scores (SDS) based on the means and standard deviations of the age-matched healthy control groups, with a higher SDS indicating better performance.

ANOVA was used to explore the interaction between neurological manifestations such as encephalopathy, seizures, MRI-detected lesions, and neurocognitive profiles. In addition, a Random Forest model was implemented to assess the relevance of neurocognitive variables in the context of learning disabilities. Data are presented as boxplots, with the interquartile range (IQR) as the box, median as the horizontal line in the box, and mean as the triangle inside the box. The ends of the boxplots extend to the most extreme data point, which is no more than 1.5 times the length of the box. Points outside this range were considered as outliers. For neurocognitive variables, the Variance Inflation Factor (VIF) was initially applied to assess multicollinearity among the variables. Variables with high VIF values were subjected to a dimensional reduction process using Principal Component Analysis (PCA).

## Results

### Participant characteristics

**Fig. 1:**
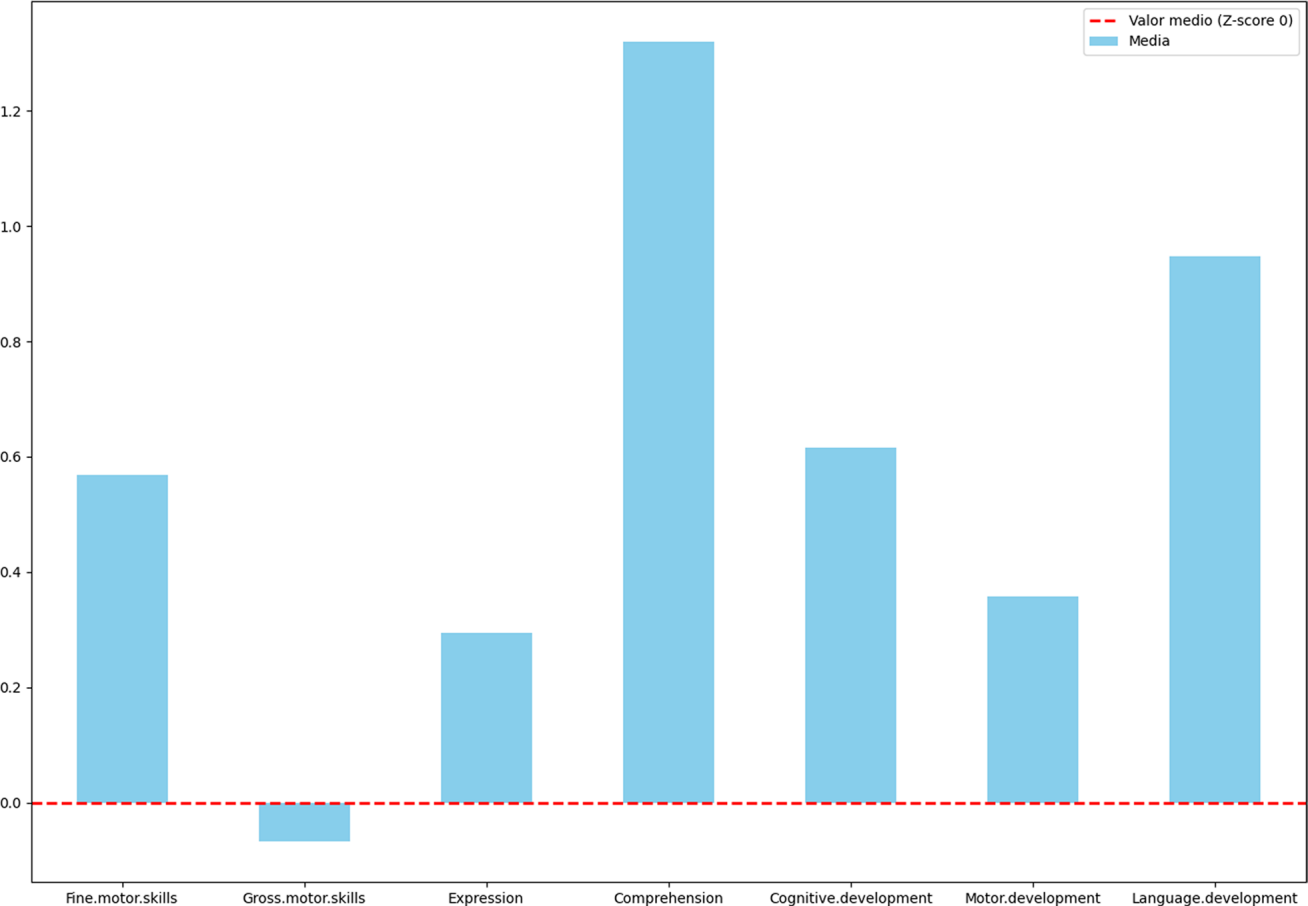
Neurocognitive Outcomes in Children Under 4 Years of Age: Description: This figure displays the cognitive performance of children under 4 years of age, focusing on domains such as gross and fine motor skills, language, and other processes evaluated using the Bayley-III scale

A total of 42 participants agreed to participate in the study, of which 41 were genetically confirmed. The median age was 48 months (IQR:31–93), most patients were Caucasian, and 59% were boys. Regarding sociodemographic variables, 59.5% of the mothers had attained a university education, and of these, 66.7% were currently in active employment. In contrast, approximately 35.7% of fathers completed university studies, and almost all of them (95.2%) were actively employed (Fig. 1).

### Neurocognitive outcomes

**Fig. 2:**
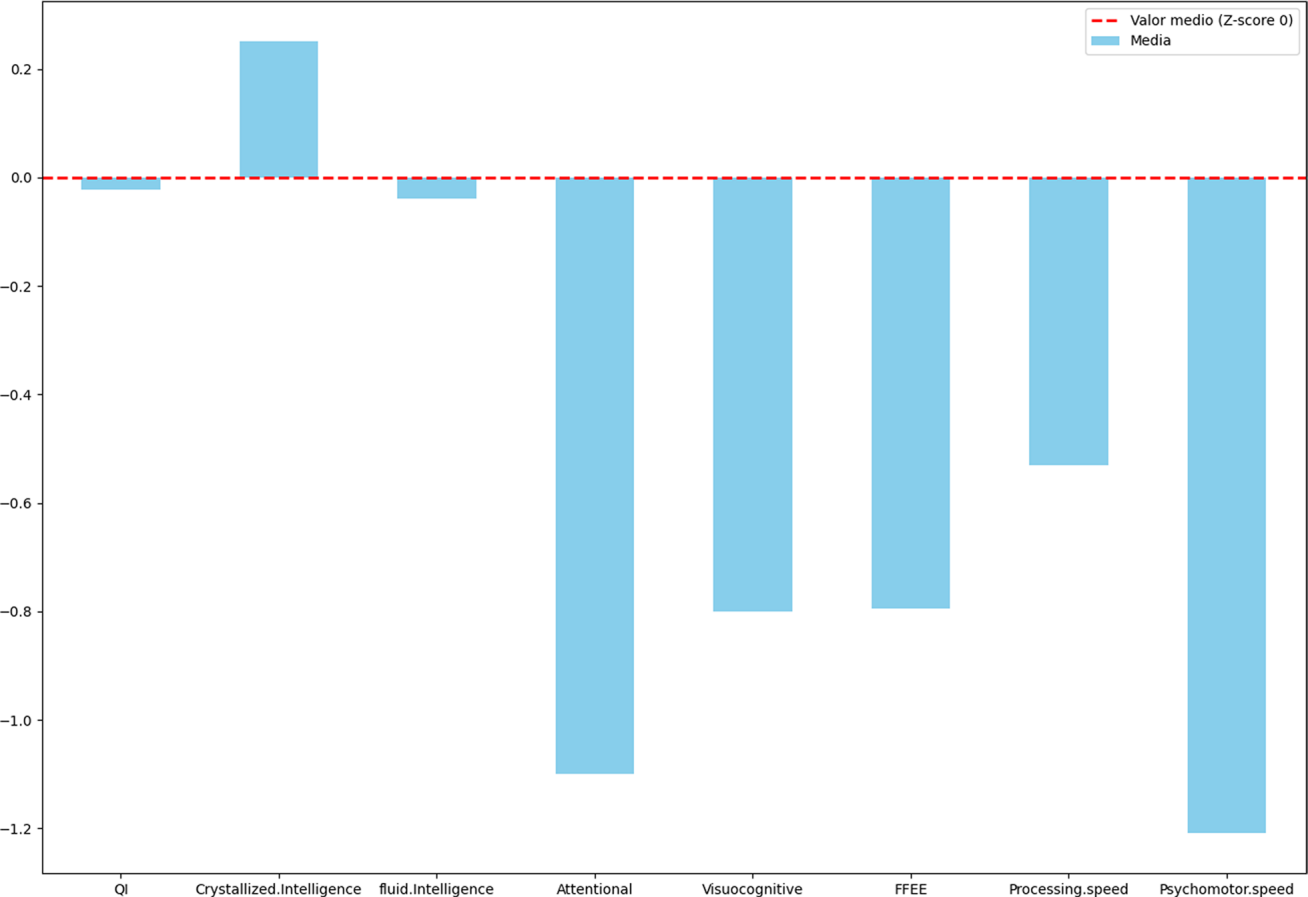
Neurocognitive Outcomes in Children Aged 4 Years and Older: Description: This figure presents the cognitive performance of children aged 4 years and older, with specific emphasis on innate and crystallized intelligence, processing speed, and executive functions

Considerable variability was observed in the sample with respect to the results obtained from psychometric tests, as shown in Table 3 and Fig. 2.

In patients under 4 years of age in terms of Bayley-III results, the mean cognition score was within the average range (0.45 ± 1.26), showing the lowest results in the gross motor subscale (− 0.35 ± 1.28). No alterations were observed in other evaluated processes, language, or fine motor skills.

In patients older than 4 years of age, the results of the intelligence tests were situated in an average profile (0.045 ± 0.62), with an innate intelligence with a z score of-0.05 ± 0.62 and crystallized intelligence with a mean z-score of 0.34 ± 0.74. In other words, both values were within the normal ranges.

Performance on psychomotor speed was generally good on the Grooved Pegboard task, although some patients showed pathological performance ranging from moderate to severe impairment. Specifically, three out of 22 participants obtained very low scores on both hands compared to their normative age group.

For attentional processes, the average score was within the normal range but close to mild deficits (z-score − 0.95 ± 0.96). There was considerable variability in performance, with deficits ranging from mild to severe deficits. A few patients scored above average (z-score = 0).

Regarding executive function and working memory, most patients exhibited moderate deficits (z-score − 1.54 ± 0.74). Only one participant scored above a z-score of 0, whereas the other participant showed severe impairment, with a z-score below − 3.

Processing speed showed an average performance within normal limits (z-score − 0.36 ± 1.34). The lowest observed score was a z-score of − 2.3, indicating moderate impairment.

In visuocognitive processes, patients had an average score close to mild deficits (z-score − 0.73 ± 0.66). There was considerable intrapersonal variability, with many patients showing deficits ranging from mild to moderate (z-score − 2). Common difficulties were found in visual perception and spatial coding, particularly in understanding abstract figures. The most severely affected patient had a z-score of − 3, indicating severe impairment.

### Learning outcomes

Data were collected from 27 out of 42 patients, as these were the ones who were enrolled in school. Among them, 92.59% required school support, as shown below.
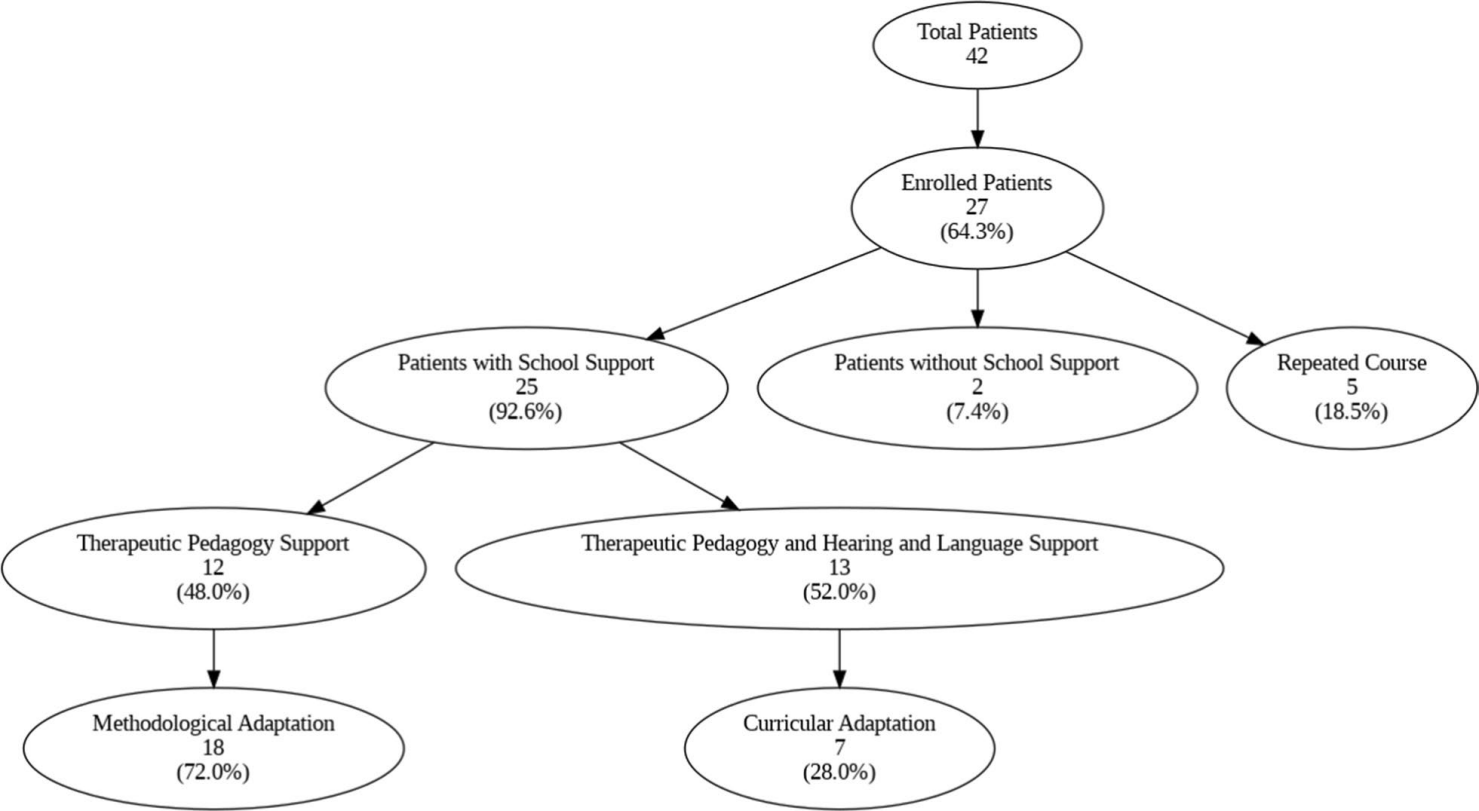


### Associations of neurocognitive functioning and their impact on learning

**Fig. 3:**
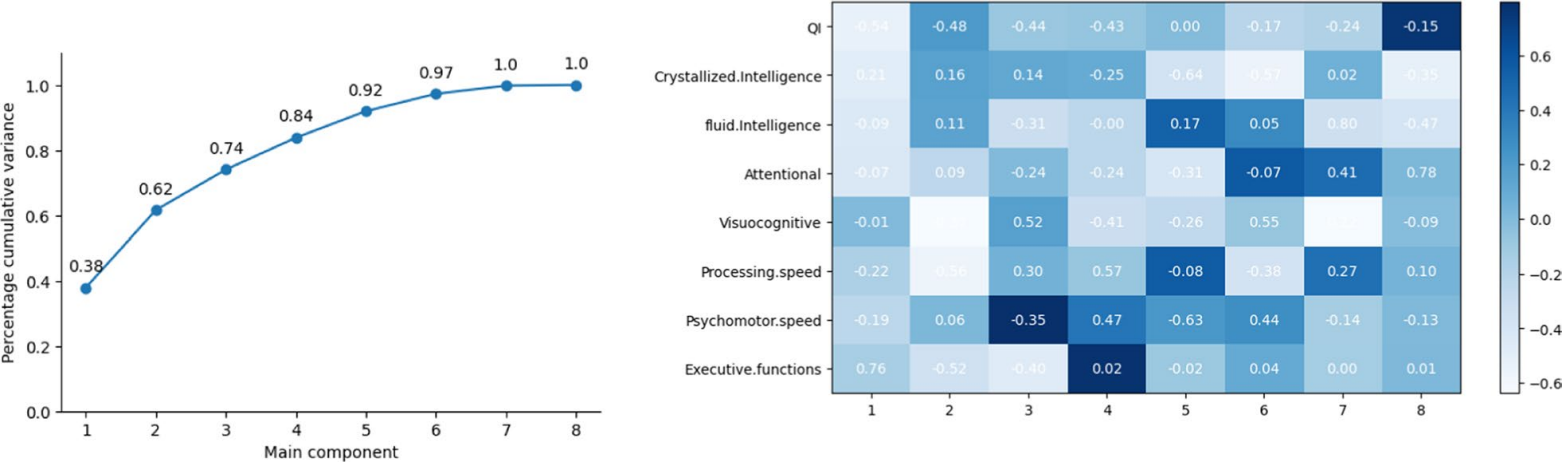
Factor Loadings in Principal Component Analysis (PCA) for Learning Impact: Description: This table provides the Factor Loadings in Principal Component Analysis (PCA), demonstrating the relationship between cognitive performance (e.g., IQ, fluid and crystallized intelligence, attention) and the need for special educational support

All the independent variables examined exceeded a VIF of 5. This high degree of interdependence suggests the need to apply dimensional reduction techniques such as PCA to distill essential information and minimize data redundancy before proceeding with further analysis. The PCA revealed that six principal components explained 97% of the total variance, justifying their selection for an efficient and non-redundant representation of the data (Fig. 3).

Regarding the main components associated with a negative impact on learning, PC1 (*p* = 0.01) was directly related to the need for special education in the school setting. The factor loadings presented in Table 2 indicate that the first principal component was strongly associated with variables related to IQ, crystallized intelligence, fluid intelligence, and attentional processes.

**Table 2:** Neurocognitive Outcomes in Children Under 4 Years of Age: Description: This figure displays the cognitive performance of children under 4 years of age, focusing on domains such as gross and fine motor skills, language, and other processes evaluated using the Bayley-III scale

Domain	Median	Q1	Q3	Mean	SD
Fine motor skills	0.3	0.3	1.5	0.57	1.52
Gross motor skills	0.3	0.5	0.35	− 0.07	1.67
Expression	0.3	0.5	2	0.29	1.84
Comprehension	1.7	0.15	2.85	1.32	1.41
Cognitive development	0.3	0.85	1.7	0.62	1.52
Motor development	0.7	0.8	1.3	0.36	1.63
Language development	0.6	0.65	2.55	0.95	1.82

**Fig. 4:**
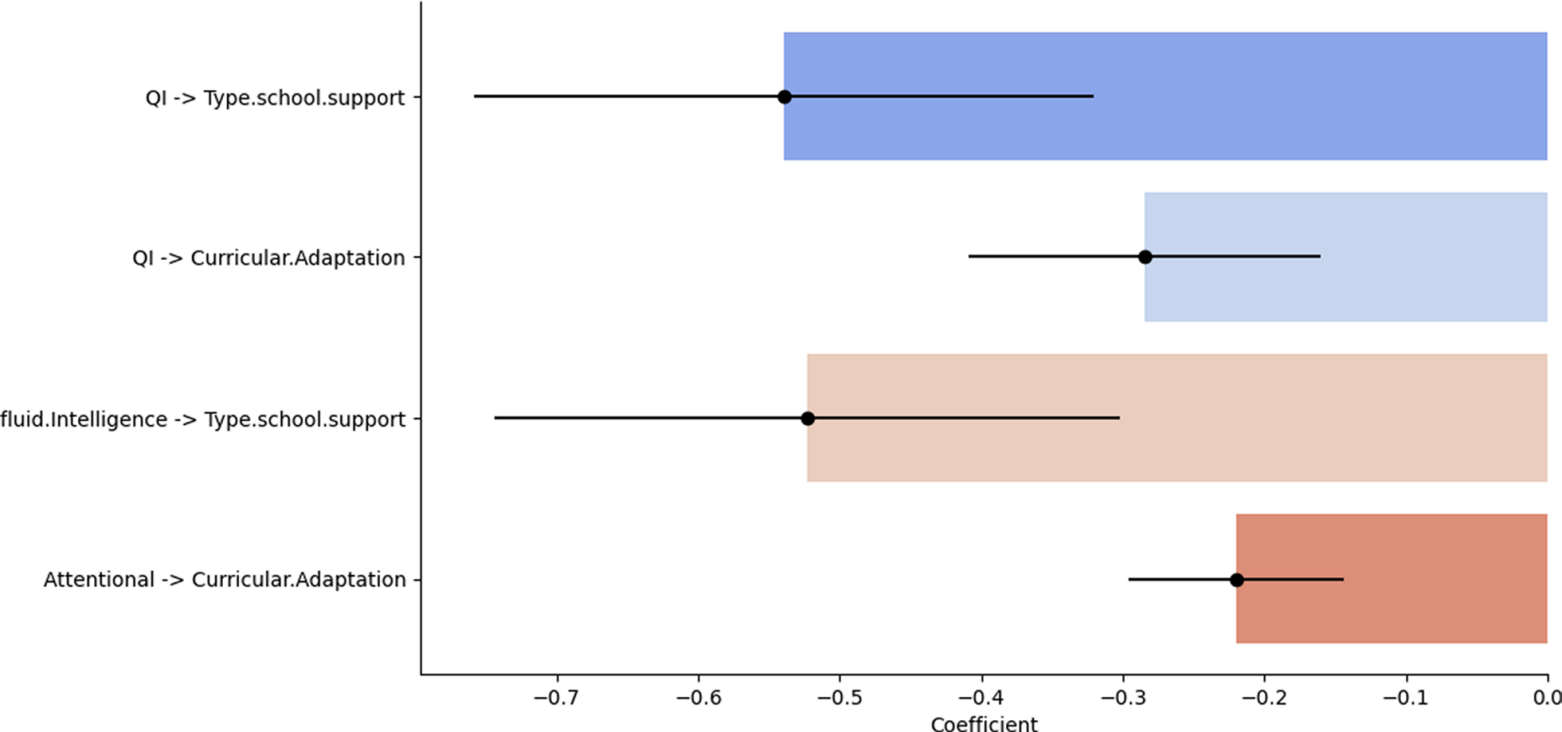
Results of individual regression analyses on neurocognitive variables. Description: This figure shows the regression analysis results illustrating the impact of IQ, fluid intelligence, and attentional capacity on learning outcomes. Higher IQ and fluid intelligence scores were significantly associated with a reduced likelihood of requiring educational support, while attentional capacity also played a key role (*p* < 0.05 for all variables)

Furthermore, we conducted individual regression analyses for each neurocognitive variable that yielded similar results. IQ, fluid intelligence, and attentional capacity significantly affected the learning outcomes. Specifically, regression analysis showed that higher IQ (coefficient − 0.5396, significance *p* < 0.05) and fluid intelligence (coefficient − 0.5225, significance *p* < 0.05) were associated with a lower likelihood of requiring educational support, whereas attentional capacity (coefficient − 0.2200, significance *p* < 0.05) was crucial (Fig. 4) (Table 3).

**Table 3:** Neurocognitive Outcomes in Children Aged 4 Years and Older: Description: This figure presents the cognitive performance of children aged 4 years and older, with specific emphasis on innate and crystallized intelligence, processing speed, and executive functions

Domain	Median	Q1	Q3	Mean	SD
Crystallized intelligence	0.3	0	0.6	0.25	0.9
Fluid intelligence	0.1	− 0.1	0.35	− 0.04	0.74
Attentional	1	− 1.65	0.5	1	1.14
Visuocognitive	0.9	− 1.35	0	0.8	0.78
Executive functions	0.9	− 1.3	0	0.8	0.77
Processing speed	0.7	− 1.25	− 0.2	0.53	1.24
Psychomotor speed	− 1.33	3	0.05	− 1.21	1.83

## Discussion

Glutaric Aciduria Type 1 (GA-1) is an inherited metabolic disorder that prevents the breakdown of certain amino acids, resulting in the accumulation of glutaric acid and its derivatives in the body [10, 18, 24, 25]. This accumulation may be particularly detrimental to the brain during crucial years of early neurocognitive development [7, 23]. Despite significant advances in our understanding of GA-1, remarkable variability in its effects and responses to treatment persists among patients. This study aimed to explore this variability in depth by analyzing a broad spectrum of neurocognitive variables in the largest cohort of Spanish patients with GA-1, thereby emphasizing the importance of a personalized approach in the treatment and management of this condition.

This study enabled a comprehensive exploration of the crucial neurocognitive variables in childhood development. The most salient findings were as follows:

GA-1 has a notably variable effect on neurodevelopment, characterized by a wide range of neurocognitive alterations that detrimentally affect learning processes. This variability aligns with the nature of inborn errors in intermediary metabolism described by Fernandez-Carvajal et al. [9] and [7, 17, 25]. Hence, these metabolic diseases can lead to severe disabilities in pediatric patients, significantly affecting their quality of life, as highlighted by Smeitink et al. [6], [4, 19, 20, 25]. The cohort utilized for this research revealed that, despite having cognitive means within normal parameters, there were patients with scores > 2 standard deviations below the population mean. This finding is particularly relevant in minority populations and in research on rare diseases to understand these impacts, develop personalized interventions, and underscore the need for continued research to better support GA-1 patients. Likewise, patients under the age of 4 years show globally adequate maturational development, with gross motor function as the only affected area, although without functional implications. These data demonstrate that disease toxicity may not affect the onset of brain development; nevertheless, neurotoxic accumulation may interfere with the development of more complex brain processes, such as cognitive, attentional, and/or executive skills [2, 6, 21].

As a result, focal alterations in neurodevelopment have been detected in patients over 4 years of age, especially in executive, attentional, and visuocognitive functions. These alterations manifest differently in GA-1 populations, underlining the heterogeneous nature of this disorder.

Remarkably, more than 60% of the sample utilized in the investigation needed adaptations and school support to keep up with learning, thus indicating that, despite effective control of their disease, they represent an at-risk group. Neurocognitive impairments associated with GA-1 significantly hinder the learning abilities of affected individuals. For example, delays in motor skills may lead to dysgraphia or, in the youngest children, difficulty making strokes at the onset of literacy learning. Deficits in language and executive functioning may affect communication and problem-solving skills as well as difficulties with inhibitory control for test-taking and even healthy peer relationships [1, 3].

This study has some limitations. One of the main limitations of our study is the heterogeneity of the samples in terms of age and disease severity. In addition, the sample size, although adequate for preliminary analyses, may limit the generalizability of our findings. These limitations should be considered when interpreting the results of this study.

## Conclusion and future research

A study of Glutaric Aciduria Type 1 (GA-1) revealed a broad spectrum of neurocognitive impairments affecting learning processes, potentially manifested through focal damage in specific areas such as executive, attentional, and visuocognitive functions. This study emphasizes the critical significance of early intervention, highlighting the necessity of personalized treatment strategies to mitigate the onset of illness and its clinical developmental effects. This underscores the importance of tailored educational support owing to the prevalence of special educational needs in the study cohort. Furthermore, it is essential to consider the relationship between the extent and type of neuronal damage and the potential cognitive outcomes when establishing the diagnosis and prognosis of the disease. On the basis of our findings, we suggest several areas for future research. It would be beneficial to further explore the relationship between the time of diagnosis and the neurocognitive and school outcomes. In addition, longitudinal studies could provide a deeper understanding of the long-term evolution of neurocognitive and school impact in GA-1 patients. Another intriguing area to explore, building upon the studies referenced in the discussion [5, 12, 21], would involve investigating the correlation between the biochemical subtype phenotype and its clinical and radiological outcomes. This analysis aimed to determine if there exists a distinction among these phenotypes that underlies a more pronounced neurological impairment, potentially elucidating the cognitive vulnerability observed within this population.

Future research and publications will also focus on analyzing cohort differences in key factors, such as the biochemical subtype—whether high or low excreter—diagnosis as a result of newborn screening, treatment prescribed, and compliance with treatment. Understanding how these variables influence findings and outcomes will be essential for improving clinical approaches and optimizing patient care.

## Data Availability

The data that support the compendium of findings of this study are not openly available in order to protect the privacy of the patients. Upon a reasonable request the data are available from the corresponding author.
